# Therapeutic potential of pycnogenol: antioxidant, anti-inflammatory, immunomodulatory, antiviral, and anticancer effects

**DOI:** 10.3389/fphar.2026.1755175

**Published:** 2026-03-09

**Authors:** Mohammad Enamul Hoque Kayesh, Michinori Kohara, Kyoko Tsukiyama-Kohara

**Affiliations:** 1 Department of Microbiology and Public Health, Faculty of Animal Science and Veterinary Medicine, Patuakhali Science and Technology University, Barishal, Bangladesh; 2 Transboundary Animal Diseases Center, Joint Faculty of Veterinary Medicine, Kagoshima University, Kagoshima, Japan; 3 Department of Microbiology and Cell Biology, Tokyo Metropolitan Institute of Medical Science, Tokyo, Japan

**Keywords:** anticancer, anti-inflammatory, antioxidant, antiviral, French maritime pine, immunomodulatory, pycnogenol

## Abstract

Pycnogenol (PYC), a standardized extract derived from the bark of the French maritime pine (*Pinus pinaster* ssp. *atlantica*), exhibits a broad spectrum of biological activities, including antioxidant, anti-inflammatory, immunomodulatory, antiviral, and anticancer effects. These effects are attributed to the rich profile of polyphenolic compounds, which confer potent antioxidant and anti-inflammatory properties. Viral infections frequently induce oxidative stress, inflammation, and immune dysregulation, thereby posing substantial challenges to global public health. Accordingly, the development of effective antiviral agents applicable across diverse viral outbreak settings remains a critical goal. PYC has demonstrated antioxidant, anti-inflammatory, and antiviral potential against several viruses, including hepatitis C virus, dengue virus, and severe acute respiratory syndrome coronavirus 2. In addition, PYC exhibited anticancer activity by modulating cell signaling pathways, inhibiting tumor cell proliferation, inducing apoptosis, and suppressing angiogenesis. However, further research and clinical validation are required to confirm its therapeutic applications. Accordingly, this review summarizes the current understanding regarding the antioxidant, anti-inflammatory, and anticancer mechanisms of PYC. Moreover, the review highlights its immunomodulatory properties to inform future antiviral and anticancer drug development and therapeutic strategies.

## 1 Introduction

Pycnogenol (PYC), a flavonoid-rich polyphenolic bark extract of the French maritime pine (*Pinus pinaster* ssp. *atlantica*), contains many key components, including procyanidins, ferulic acid, taxifolin, catechin, and caffeic acid (Lee et al., 2010). PYC exhibits a wide range of pharmacological properties, largely attributed to its potent antioxidant and anti-inflammatory activities.

Chronic inflammation and oxidative stress are central contributors to the pathogenesis of numerous diseases, including type 2 diabetes, cardiovascular diseases, and various liver conditions (Nattagh-Eshtivani et al., 2022). The diverse bioactive components of PYC enhance endogenous antioxidant defense systems and modulate inflammatory responses (Packer et al., 1999; Kim et al., 2000; Chovanová et al., 2006; Ozer Sehirli et al., 2009; Kim et al., 2011; Peng et al., 2012; Uhlenhut and Högger, 2012; Muchová et al., 2014; Cotellese et al., 2021; Nattagh-Eshtivani et al., 2022; Liu et al., 2024). Specifically, PYC reduces lipid peroxidation and protein carbonylation, thereby protecting cellular components from oxidative damage (Sivonová et al., 2006; Ansari et al., 2008). PYC also inhibits the activation of nuclear factor-kappa-light-chain-enhancer of activated B cells (NF-κB), a key regulator of inflammatory and immune responses, thereby contributing to its anti-inflammatory and immunomodulatory effects (Grimm et al., 2006; Fan et al., 2015).

In clinical and experimental settings, PYC reduces the cardiovascular risk in patients with type 2 diabetes (Zibadi et al., 2008), exerts anticancer activity (Becit and Aydin, 2020), and improves skin health via photoprotection, thereby reducing hyperpigmentation and enhancing skin barrier function and extracellular matrix homeostasis (Grether-Beck et al., 2016).

Furthermore, PYC modulates inflammatory gene expression in human leukocytes by inhibiting 5-lipoxygenase and cyclooxygenase (COX)-2, with a compensatory upregulation of COX-1 gene expression (Canali et al., 2009). Given that COX2 overexpression has been implicated in various cancers, including colorectal and prostate carcinomas (Sano et al., 1995; Yoshimura et al., 2000; Hyde and Missailidis, 2009), modulating this pathway underscores the promising potential of PYC as an anticancer agent.

In addition to its anti-inflammatory and antioxidant effects, PYC demonstrates diverse therapeutic effects. Specifically, PYC improves endothelial function, microcirculation, and inflammatory marker profiles in patients recovering from symptomatic coronavirus disease-2019 (COVID-19) (Belcaro et al., 2022). PYC also exhibits broad-spectrum antimicrobial activity against bacteria, yeast, and fungi at low concentrations (Torras et al., 2005) and prevents recurrent urinary tract infections and interstitial cystitis (Ledda et al., 2021). Furthermore, PYC and its phenolic constituents improve gut health (Sánchez-Moya et al., 2024).

Given the increasing threat of emerging and re-emerging viral infections (Luo and Gao, 2020; Baker et al., 2022), the development of effective antiviral agents remains a pressing challenge. PYC has shown promising antiviral activity in a murine model of myocarditis, where it markedly reduced viral replication and cardiac inflammation (Matsumori, 2007). Additional studies have further supported its antiviral efficacy (Matsumori et al., 2007; Ezzikouri et al., 2015; Weichmann and Rohdewald, 2024), highlighting its potential as an alternative or adjunct antiviral therapy. However, more robust clinical investigations are required to confirm these findings and establish therapeutic applications (Mármol et al., 2019). Accordingly, this review aims to comprehensively explore the antioxidant, anti-inflammatory, immunomodulatory, anticancer, and antiviral effects of PYC, with a particular focus on its potential in the development of novel therapeutic strategies, including antiviral interventions.

## 2 Antioxidative effects of PYC

PYC exhibits potent antioxidant properties by effectively scavenging reactive oxygen and nitrogen species—including superoxide (·O_2_^−^), nitric oxide (NO·), peroxynitrite (ONOO^−^), and hydroxyl radicals (·OH)—thereby counteracting oxidative stress induced by excessive reactive oxygen species (ROS) production (Kim et al., 2008). PYC polyphenolic constituents, including procyanidins, catechins, and phenolic acids, effectively scavenge free radicals and enhance endogenous antioxidant defense systems, such as superoxide dismutase (SOD) and glutathione (GSH). Two major metabolites of PYC, namely δ-(3,4-dihydroxyphenyl)-γ-valerolactone (M1) and δ-(3-methoxy-4-hydroxyphenyl)-γ-valerolactone, strongly inhibit matrix metalloproteinases (MMP-1, MMP-2, and MMP-9) activity, thereby inhibiting oxidative stress (Grimm et al., 2004). In addition, M1 scavenges superoxide radicals and exhibits potent antioxidant activity (Grimm et al., 2004). Following ozone exposure, PYC enhanced the levels of endogenous antioxidants (uric acid and ascorbic acid) and reduced reactive nitrogen species in the airways, suggesting that its protective effects involve strengthening cellular antioxidant defenses to mitigate ozone-induced oxidative damage (Lee et al., 2013). Thioredoxin reductase (TrxR) and GSH peroxidase (GPx) are typically upregulated in response to oxidative stress as part of the cellular antioxidant defense system (Ren et al., 2017; Benhar, 2018). Notably, PYC treatment reduced the activity of both TrxR and GPx, thereby decreasing oxidative stress (Gandin et al., 2009). Methotrexate (MTX), a widely used chemotherapeutic agent, induces cytotoxic effects in both cancerous and healthy tissues, largely through oxidative stress. PYC has been shown to attenuate MTX-induced oxidative damage, reducing its off-target toxicities (Abdelaziz et al., 2020; Al-Abkal et al., 2022).

Following sepsis induction in Wistar albino rats, PYC treatment decreased malondialdehyde levels, increased GSH levels, and enhanced SOD and GPx activities. This revealed its antioxidative role in reducing oxidative stress and contributing to the inhibition of melanin synthesis by modulating the GSH redox balance (Kim et al., 2008; Taner et al., 2014).

*In vitro*, PYC exhibits antioxidant effects during adipogenesis by downregulating the mRNA expression of pro-oxidant enzymes, such as nicotinamide adenine dinucleotide phosphate hydrogen oxidase 4 and glucose-6-phosphate dehydrogenase, while simultaneously upregulating antioxidant enzymes, including Cu/Zn-SOD, Mn-SOD, GPx, and GSH reductase (Lee et al., 2012). These actions collectively reduce ROS production and lipid accumulation in adipocytes. Moreover, *ex vivo* studies show that PYC acts synergistically with lutein to reduce oxidative stress in porcine retinal homogenates (Nakanishi-Ueda et al., 2006).

In a study using *Escherichia coli* mutants, PYC induced the expression of key antioxidant enzymes such as MnSOD and CuZnSOD, particularly under oxidative stress, suggesting a potential antioxidative role (Youm and Kim, 2003). However, these findings also indicate that the effects of PYC may be context-dependent and may exhibit pro-oxidant properties under certain conditions, warranting further evaluation (Youm and Kim, 2003).

Similarly, PYC mitigated oxidative stress and inflammation induced by titanium dioxide nanoparticles in both murine lung tissue and human airway epithelial cells (Lim et al., 2024). This antioxidative effect was attributed to the restoration of GSH levels, upregulation of antioxidant enzymes, and suppression of thioredoxin interacting protein, a key mediator of oxidative stress and cytotoxicity (Lim et al., 2024).

**FIGURE 1:**
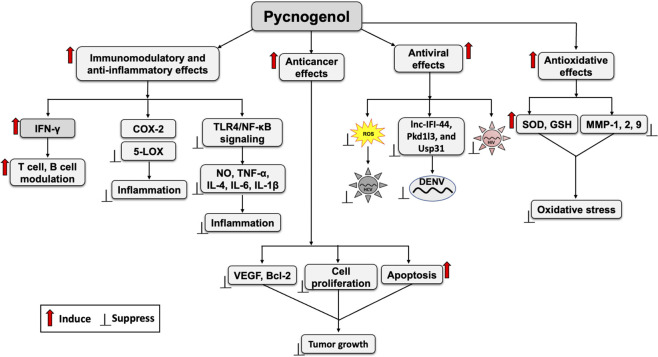
Effects of pycnogenol (PYC) on oxidative stress, host immunity, inflammation, viral infection, and cancer. Potential target pathways of PYC contributing to its antioxidative, immunomodulatory, anti-inflammatory, antiviral, and anticancer effects are indicated. Red arrows indicate stimulation and black blunt-ended arrows indicate suppressive effects. Abbreviations: IFN-γ, interferon-γ; COX-2, cyclooxygenase-2; 5-LOX, 5-lipoxygenase; TLR4, toll-like receptor 4; NF-κB, nuclear factor κ-light-chain-enhancer of activated B cells; NO, nitric oxide; TNF-α, tumor necrosis factor α, IL, interleukin, VEGF, vascular endothelial growth factor; Bcl-2, B-cell lymphoma 2; Usp13, ubiquitin-specific protease 13; ROS, reactive oxygen species; MMP, matrix metalloproteinases; SOD, superoxide dismutase; GSH, glutathione; lnc-IFI-44, long non-coding RNA interferon-induced protein 44; Pkd1l3, polycystic kidney disease 1-like 3; HCV, hepatitis C virus; HIV, human immunodeficiency virus; DENV, dengue virus.

In a randomized clinical trial involving pediatric patients with attention deficit hyperactivity disorder, PYC treatment produced a modest increase in catalase activity, suggesting potential antioxidant effects (Weyns et al., 2022). However, consistent evidence supporting the significant antioxidant benefits compared with placebo or methylphenidate has not yet been established (Weyns et al., 2022). Overall, PYC exhibits robust antioxidant activity (Figure 1) in diverse contexts and experimental models. Through these mechanisms, it contributes substantially to the maintenance of redox homeostasis, particularly during inflammatory and immune responses.

## 3 Immunomodulatory and anti-inflammatory effects of PYC

The immunomodulatory effects of PYC have been demonstrated extensively (Park et al., 2000; Grimm et al., 2006; Ko et al., 2017; Weichmann and Rohdewald, 2024). In an *in vitro* study, Bito et al. (2000) investigated the effect of PYC on interferon (IFN)-γ-induced intercellular adhesion molecule-1 (ICAM-1) expression and found that PYC downregulated T-cell adhesion to human keratinocytes by inhibiting ICAM-1 expression. PYC also suppresses IFN-γ-mediated activation of the signal transducer and activator of transcription-1, which may regulate inducible ICAM-1 transcription (Bito et al., 2000).

Moreover, the toll-like receptor (TLR)-mediated immunomodulatory effects of PYC have been investigated (Verlaet et al., 2019). PYC exerts anti-inflammatory effects by inhibiting the TLR4– nuclear factor κ-light-chain-enhancer of activated B cells (NF-κB) pathway (Verlaet et al., 2019). Considering that intact, non-metabolized PYC acts as a dose-dependent agonist of TLR1/2 and TLR2/6 (Verlaet et al., 2019), other TLR signaling pathways may also contribute to its functions. In addition, PYC suppresses adipose differentiation-related protein, which is involved in atherosclerosis development and highly expressed in macrophages (Gu et al., 2008), by inhibiting activator protein-1 (AP-1) and NF-κB activities, thereby reducing atherosclerosis (Gu et al., 2008). PYC further inhibits atherosclerosis by regulating lipid metabolism via the TLR4–NF-κB pathway (Luo et al., 2015). However, the involvement of other TLRs in PYC function and the mechanisms underlying these inhibitory effects remain unclear.

In an *in vitro* study on microglial cells, PYC dose-dependently decreased the release of nitric oxide, tumor necrosis factor-α (TNF-α), interleukin (IL)-6, IL-1β, ICAM-1, and perilipin 2, thereby exhibiting anti-inflammatory activity (Fan et al., 2015). Furthermore, PYC effectively alleviated inflammation by inhibiting the NF-κB and AP-1 pathways (Fan et al., 2015), highlighting its therapeutic potential against inflammatory condition.

*Ex vivo* analysis of plasma from volunteers following PYC administration revealed moderate inhibition of COX-1 and COX-2 activity, indicating potential anti-inflammatory and platelet-inhibitory effects (Schäfer et al., 2006).

PYC modulated cytokine secretion by decreasing in T-helper (Th)1 cytokine levels and increasing Th2 cytokine levels and partially recovered T- and B-cell dysfunction in a mouse model (Lee et al., 2005). It also increased IL-2 and IFN-γ secretion while decreasing IL-4, IL-6, and TNF-α levels, thereby helping to prevent immune dysfunction in CS7BL/6 mice with acquired immunodeficiency syndrome (Lee et al., 2005).

In a murine model, PYC demonstrated neuroprotective effects following traumatic brain injury, characterized by a reduction in the proinflammatory cytokines IL-6 and TNF-α (Scheff et al., 2013). Transforming growth factor (TGF)-β family members are potent regulatory cytokines that influence multiple cell types and mediate both proinflammatory and anti-inflammatory responses (Schon and Weiskirchen, 2014). PYC modulates TGF-β1 expression and the phosphorylation of SMAD (mothers against decapentaplegic homolog) family member 2/3, effectively suppressing these signaling pathways (Ko et al., 2017). Furthermore, a randomized, double-blind, placebo-controlled trial in humans demonstrated that PYC supplementation markedly reduced the levels of key inflammatory biomarkers, including salivary MMP-8 and serum IL-6, supporting its anti-inflammatory effects (Bayer et al., 2025). Similarly, PYC decreased circulating levels of C-reactive protein (CRP), a hepatic pentraxin that rises in response to tissue injury, infection, and inflammation (Ansar and Ghosh, 2013; Braig et al., 2017; Sproston and Ashworth, 2018), further highlighting its anti-inflammatory potential (Nikpayam et al., 2018).

Collectively, these reports reveal the potent anti-inflammatory and immunomodulatory effects of PYC (Figure 1), warranting further clinical trials to evaluate its therapeutic efficacy against various infections, including viral infections that induce host inflammation and immune dysregulation.

## 4 Antiviral effects of PYC

PYC exhibits antiviral activity against a broad range of viruses, including hepatitis C virus (HCV), dengue virus (DENV), classical swine fever virus (CSFV), and human immunodeficiency virus (HIV), among others (Figure 1) (Ezzikouri et al., 2015; Ide et al., 2022; Hossain et al., 2024). Its antiviral effects appear to be multifaceted, encompassing both direct inhibition of viral replication and modulation of host factors that regulate the viral replication cycle and immune responses.

Substantial insight into PYC’s antiviral potential has emerged from *in vitro* and *in vivo* studies of HCV. Using HCV replicon systems and chimeric mice models infected with HCV genotypes 2a and 1b, PYC was shown to enhance the antiviral efficacy of ribavirin, IFN, and telaprevir, either synergistically or additively (Ezzikouri et al., 2015). Notably, combined treatment with PYC (40 μg/kg) and pegylated (PEG)-IFN (30 μg/kg) significantly reduced HCV RNA levels after 14 days, outperforming either monotherapy alone (Ezzikouri et al., 2015). These findings suggest that PYC can potentiate existing antiviral regimens, particularly by facilitating the elimination of resistant HCV mutants, although the precise molecular mechanisms remain incompletely understood.

One key mechanism underlying PYC’s antiviral activity is its ability to modulate oxidative stress. PYC treatment reduces ROS production in HCV replicon cell lines, thereby limiting viral replication. This antioxidant effect also enhances the activity of direct-acting antivirals such as telaprevir, especially in telaprevir-resistant replicon cells (Ezzikouri et al., 2015). HCV infection and type 2 diabetes are known to have a bidirectional relationship, with each condition increasing susceptibility to the other (Grimbert et al., 1996; Mehta et al., 2003; Romero-Gómez et al., 2005; Hammerstad et al., 2015). In this context, PYC supplementation has been proposed to mitigate HCV-associated type 2 diabetes (Ezzikouri et al., 2016), although this hypothesis requires validation in clinical studies. Overall, available evidence supports a role for PYC in enhancing antiviral efficacy and reducing viral resistance in HCV infection.

Beyond HCV, PYC also modulates host factors implicated in viral replication and immune regulation, including long non-coding IFN-induced protein-44 (lnc-IFI-44), polycystic kidney disease 1-like 3 (Pkd1l3), and ubiquitin-specific peptidase 31 (Usp31) (Ide et al., 2022). Silencing of these host factors inhibits DENV replication, suggesting that PYC’s antiviral effects against DENV, and potentially other viruses, may be mediated through host-directed mechanisms (Hossain et al., 2024). However, the detailed molecular mechanisms by which these factors suppress DENV replication remain to be elucidated. Consistent with this mechanism, PYC reduces viral loads of all four DENV serotypes in a dose-dependent manner, likely by interfering with late stages of infection such as viral maturation and secretion (Hossain et al., 2024). PYC also inhibits CSFV replication in a dose-dependent fashion, underscoring its broad-spectrum antiviral potential (Ide et al., 2022).

PYC has also been reported to inhibit HIV-1 entry and replication (Feng et al., 2008), indicating antiviral activity at early stages of the viral life cycle, although its precise molecular targets remain unclear. Consistent with these findings, PYC has been shown to alleviate common cold symptoms, shorten illness duration, and reduce complications associated with rhinovirus infections (Belcaro et al., 2014). These effects suggest that PYC may act, at least in part, by modulating host immune defenses and attenuating virus-induced inflammation. Supporting this notion, procyanidin-rich compounds extracted from pine cones have been reported to inhibit herpes simplex virus type 1 and type 2 replication in African green monkey kidney cells and human adenocarcinoma cells (Fukuchi et al., 1989). Collectively, these observations indicate that PYC may exert broad-spectrum antiviral activity by targeting viral entry, replication, and host-pathogen interactions, highlighting the need for further mechanistic studies, particularly in the context of herpes simplex virus infection.

Importantly, PYC’s antiviral activity extends beyond direct viral inhibition to include complementary host-directed effects. By modulating oxidative stress and inflammatory signaling, PYC targets key determinants of disease severity and treatment outcomes. For example, oxidative stress exacerbates lung inflammation in respiratory viral infections such as severe acute respiratory syndrome-coronavirus-2 (SARS-CoV-2) and influenza A virus infections (Khomich et al., 2018; Xie et al., 2024). Given that PYC suppresses ROS production in HCV infection (Ezzikouri et al., 2015), it may also have therapeutic potential in managing ROS-mediated inflammation in other viral infections, including influenza A and SARS-CoV-2.

Severe SARS-CoV-2 infection is characterized by cytokine storm, coagulopathy, and endothelial inflammation driven by excessive immune activation. In this context, the anti-inflammatory properties of PYC, particularly its inhibition of the TLR4–NF-κB signaling pathway, represent a critical link between redox regulation and immune modulation (Verlaet et al., 2019). A human clinical study further demonstrated that PYC reduces systemic inflammation and improves vascular and endothelial function (Weichmann and Rohdewald, 2020). Given that TLR2 and TLR4 play central roles in promoting innate immune overactivation during SARS-CoV-2 infection (Kayesh et al., 2021; Zhao et al., 2021; Zheng et al., 2021), PYC may exert antiviral and protective effects through coordinated suppression of oxidative stress and TLR-mediated inflammatory signaling (Ye et al., 2020; Weichmann and Rohdewald, 2020). Further studies are warranted to elucidate its effects on TLR2- and TLR4-driven responses in SARS-CoV-2 infection.

Recent translational preclinical studies have explored the use of PYC-based nanocomposites as a strategy to enhance the delivery of antiviral agents, including molnupiravir, for the treatment of COVID-19 (El-Shafai et al., 2024). Although these findings are promising, further validation is required before clinical application. An ongoing clinical trial is currently evaluating the effects of PYC supplementation on health outcomes in post-COVID-19 patients (Radtke et al., 2024) (ClinicalTrials.gov: NCT05890534). Moreover, a clinical study demonstrated that daily administration of 200 mg PYC for 12 weeks reduces circulating hepatitis B virus (HBV) levels and improves hepatic function, suggesting potential therapeutic applicability in HBV infection (Matsumori, 2022).

Overall, PYC’s antiviral effects are likely mediated by a combination of direct viral inhibition and host-directed mechanisms, including modulation of oxidative stress and immune responses. Although the precise active compounds and molecular pathways responsible for these effects remain to be fully defined, current evidence supports PYC’s potential as a safe and effective candidate for the management of viral infections. Future studies should focus on identifying its active constituents, elucidating detailed mechanisms of action, and validating its efficacy in well-designed clinical trials. A summary of PYC’s effects on oxidative stress, immune responses, inflammation, viral infections, and cancer is provided in Table 1.

**TABLE 1:** Effects of pycnogenol on oxidative stress, immune response, inflammation, viral infections, and cancer.

Disease/Condition target	Observed effect	Model	Mechanism of action	References
Cognitive aging, attention-deficit hyperactivity disorder	Antioxidative and anti-inflammatory	*In vivo* (humans)	Mitigates oxidative stress by scavenging free radicals, protecting DNA from oxidation, enhancing antioxidant enzyme production, and stabilizing endogenous antioxidants (vitamins C and E and glutathione)	Simpson et al. (2019), Weyns et al. (2022)
Traumatic brain injury	Antioxidative and anti-inflammatory	*In vivo* (human and mouse model)	Inhibits inflammatory processes and suppresses lipid peroxidation	Anthonymuthu et al. (2018), Malekahmadi et al. (2020), Fesharaki-Zadeh (2022)
Immune dysfunction	Immunomodulatory	*In vivo* (mice)	Increased IL-2 and IFN-γ secretion. Decreased IL-4, IL-6, and TNF-α secretion	Lee et al. (2005)
Skin inflammation	Anti-inflammatory	*In vitro*	Downregulated ICAM-1 expression	Bito et al. (2000)
Cytokine storm in SARS-CoV-2 infection	Anti-inflammatory	*In vitro,* *in* *vivo* (humans)	Improved vascular and endothelial function. Inhibited TLR4–NF-κB pathway	Verlaet et al. (2019), Weichmann and Rohdewald (2020)
Inflammation	Anti-inflammatory	*In vitro*	Suppressed TLR signaling. Induced IL-10 secretion	Verlaet et al. (2019)
CRP	Anti-inflammatory	*In vivo* (humans)	Decreased CRP levels	Nikpayam et al. (2018)
Brain disease	Anti-inflammatory	*In vitro*	Inhibited NF-κB/AP-1 activation. Decreased NO, TNF-α, IL-6, an IL-1β release	Fan et al. (2015)
Traumatic brain injury	Anti-inflammatory	*In vivo* (rat)	Reduces IL-6 and TNF-α	Scheff et al. (2013)
HCV infection	Antiviral	*In vitro*, *in vivo* (chimeric mice)	The exact mechanism is unknown. Reduces ROS production	Ezzikouri et al. (2015)
Dengue virus infection	Antiviral	*In vitro*, *in vivo* (mice)	Inhibited the expression of lnc-IFI-44, Pkd1l3, and Usp31 and reduced viral replication	Hossain et al. (2024)
Cancer	Anticancer	*In vitro*	Enhanced caspase-independent or caspase 3-dependent apoptosis and stabilized pro-apoptotic Bak protein	Huang et al. (2005), Yang et al. (2014)
Cancer	Anticancer	*In vitro*, *ex vivo*	Induced apoptosis	Harati et al. (2015)

SARS-CoV-2, severe acute respiratory syndrome-coronavirus-2; CRP, C-reactive protein; HCV, hepatitis C virus; IL, interleukin; IFN-γ, interferon-γ; TNF-α, tumor necrosis factor-α; ICAM-1, intercellular adhesion molecule-1; TLR, toll-like receptor; NF-κB, nuclear factor kappa-light-chain-enhancer of activated B cells; AP-1, activator protein 1; NO, nitrogen monoxide; ROS, reactive oxygen species; lnc-IFI-44, long non-coding IFN-induced protein-44; Pkd1l3, polycystic kidney disease 1-like 3; Usp31, ubiquitin-specific peptidase 31.

## 5 Anticancer effects of PYC

Anticancer drugs remain essential in cancer treatment because they directly or indirectly induce DNA damage, leading to cellular senescence or apoptosis through the inhibition of cancer cell proliferation (Reuvers et al., 2020). PYC has demonstrated substantial anticancer potential in various *in vitro* and *in vivo* models, supporting its role as a natural chemopreventive and therapeutic agent.

Multiple studies indicate that apoptosis induction is a primary mechanism underlying the anticancer activity of PYC. In MC-3 human mucoepidermoid carcinoma cells, PYC induced caspase-independent apoptosis and enhanced the stability of the pro-apoptotic Bak protein, suggesting its therapeutic relevance in salivary gland carcinomas (Yang et al., 2014). Similarly, PYC exhibited selective cytotoxicity toward ovarian cancer cell lines (PA-1 and TOV-21G), markedly reducing cell viability, likely through apoptotic pathways (Buz’zard and Lau, 2004). In human leukemia cell lines, PYC inhibited cell proliferation and induced apoptosis in a dose- and time-dependent manner, primarily via caspase-3 activation. Additionally, PYC promoted differentiation in HL-60 cells, highlighting its potential chemopreventive and chemotherapeutic utility in hematological malignancies (Huang et al., 2005). *Ex vivo* studies further support the pro-apoptotic effects of PYC. Plasma obtained from healthy adults following a single 300 mg oral dose of PYC induced pronounced apoptotic cell death in human fibrosarcoma (HT1080) cells, whereas plasma from untreated individuals showed no such effect (Harati et al., 2015).

Beyond direct induction of apoptosis, PYC modulates key molecular pathways involved in cancer cell survival and migration. In MCF-7 breast cancer cells, PYC exerted strong pro-apoptotic and anti-migrative effects, characterized by reduced cell viability, chromatin condensation, and downregulation of genes such as B-cell lymphoma 2, vascular endothelial growth factor (VEGF)/fibroblast growth factor, and p53. These findings suggest that modulation of VEGF-related signaling pathways contributes to the anticancer effects of PYC (Somesh et al., 2024). Consistently, microarray analysis revealed that PYC altered the expression of several apoptosis- and adhesin-related genes, including Janus kinase 1, dual specificity phosphatase 1, ras homolog family member A, laminin γ1, fibronectin 1, catenin alpha 1, and integrin subunit beta 1. This gene expression profile supports PYC’s role as a systemic pro-apoptotic agent with potential utility in combination cancer therapies (Harati et al., 2015).

PYC also exhibits indirect anticancer effects through its antioxidant properties and protection against DNA damage. In preclinical studies*,* PYC conferred dose-dependent protection against gamma irradiation-induced lung damage in rats by enhancing antioxidant enzyme activity and reducing oxidative stress markers. A dose of 300 mg/kg substantially mitigated histopathological damage and DNA fragmentation in lung tissue (Tan et al., 2022). Moreover, topical application of PYC delayed the onset and reduced the incidence and multiplicity of UV-induced skin tumors in mice. This chemopreventive effect was dose-dependent and associated with decreased UV-induced inflammation and immunosuppression, highlighting its potential as a post-exposure photoprotective and anticarcinogenic agent (Sime and Reeve, 2004).

In addition to its direct anticancer mechanisms, PYC attenuates MTX-induced toxicity in vital organs by reducing oxidative damage, inflammation, and histological damage. These cytoprotective effects suggest that PYC may serve as a beneficial adjuvant to enhance the safety and tolerability of conventional cancer therapies (Al-Abkal et al., 2022). Although PYC is widely recognized for its antioxidant and anti-inflammatory properties that contribute to cancer prevention, it is not currently classified as a standard anticancer drug. Therefore, further mechanistic *in vivo* studies and well-controlled clinical trials are required to validate its efficacy and safety in cancer treatment. The overall anticancer effects of PYC are summarized in Figure 1. In addition, a consolidated overview of the biological effects of PYC, categorized by study type and quality of evidence, is provided in Table 2.

**TABLE 2:** Evidence for pycnogenol effects categorized by study type and quality.

Effect category	Study type	Quality	Key findings	References
Antioxidative	*In vitro*	High	Scavenges ROS/RNS, enhances SOD/GSH, inhibits MMPs	Kim et al. (2008), Grimm et al. (2004)
	*In vivo*	Moderate	Reduces oxidative stress in rat lung or liver tissues	Tan et al. (2022), Abdelaziz et al. (2020)
	Clinical	Moderate	Increased catalase activity, improved antioxidant status	Weyns et al. (2022)
Anti-inflammatory/Immunomodulatory	*In vitro*	High	Suppresses NF-κB, cytokines, ICAM-1, TLR4 signaling	Fan et al. (2015), Luo et al. (2015), Verlaet et al. (2019)
Antiviral	*In vitro*	High	Inhibits HCV, DENV, HIV replication; modulates host antiviral factors	Feng et al. (2008), Ezzikouri et al. (2015), Hossain et al. (2024)
	Translational/Preclinical	Preliminary	Enhances antiviral delivery via nanocomposites	El-Shafai et al. (2024)
	Clinical	Preliminary	Reduces HBV viral load	Matsumori (2022)
Anticancer	*In vitro*	High	Induces apoptosis, inhibits proliferation, regulates VEGF and p53 pathways	Harati et al. (2015), Somesh et al. (2024)
	*Ex vivo*	Preliminary	Plasma from treated humans induces cancer cell apoptosis	Harati et al. (2015)
	*In vivo* (murine model)	Moderate	Reduces UV-induced skin tumors; protects against gamma irradiation-induced lung damage	Sime and Reeve (2004), Tan et al. (2022)

ROS, reactive oxygen species; RNS, reactive nitrogen species; SOD, superoxide dismutase; GSH, glutathione; MMPs, matrix metalloproteinases; NF-κB, nuclear factor kappa-light-chain-enhancer of activated B cells; ICAM-1, intercellular adhesion molecule-1; TLR4, toll-like receptor 4; HCV, hepatitis C virus; DENV, dengue virus; HIV, human immunodeficiency virus; HBV, hepatitis B virus; VEGF, vascular endothelial growth factor; p53, tumor protein p53.

## 6 Discussion

Although the bioavailability of PYC has not yet been fully characterized, low-molecular-weight constituents, such as catechin, caffeic acid, ferulic acid, and taxifolin are readily absorbed from the small intestine and can be detected in the bloodstream within 30 min post-ingestion (Bayer and Högger, 2024). In contrast, procyanidin oligomers and polymers undergo microbial degradation in the large intestine to produce smaller bioavailable metabolites. Moreover, prolonged presence of catechin and ferulic acid has been observed following administration (Bayer and Högger, 2024).

PYC exhibited notable antioxidant effects in a Parkinson’s disease mouse model, enhancing motor function and reducing depression-like behavior. These effects were associated with increased nuclear factor erythroid 2-related factor 2 (Nrf2) gene expression, suggesting involvement of both antioxidant and anti-inflammatory mechanisms (Jafari et al., 2022). Consistently, other murine studies have shown that PYC exerts neuroprotective effects by suppressing oxidative stress and inflammatory responses while enhancing overall antioxidant capacity (Icel et al., 2018; Nayak et al., 2025). Many viral infections, including those caused by HCV, HBV, influenza A virus, and Zika virus, modulate the host antioxidant defense system to induce oxidative stress, thereby promoting viral replication (Kayesh et al., 2025). Given its antioxidant properties, PYC may counteract this process by reducing oxidative stress-induced viral replication. PYC may also enhance antiviral defenses by inducing antioxidant proteins, such as Mn-SOD, which can inhibit the replication of viruses, such as HIV and HCV by interacting with viral proteins or virus-restrictive cellular changes (Feng et al., 2008; Ezzikouri et al., 2015). Mechanistically, PYC’s polyphenolic constituents, including procyanidins and catechins, scavenge reactive oxygen and nitrogen species, including superoxide, nitric oxide, peroxynitrite, and hydroxyl radicals, while modulating key cellular antioxidant and inflammatory pathways. PYC activates Nrf2-dependent transcription, increasing antioxidant defenses such as GSH and SOD, and suppresses ROS-sensitive NF-κB signaling, thereby reducing oxidative stress and associated pro-inflammatory responses (Grimm et al., 2004; Kim et al., 2008; Ngo and Duennwald, 2022). These coordinated antioxidant and immunomodulatory actions may further enhance the efficacy of direct-acting antivirals against viral replication (Ezzikouri et al., 2015).

TLR4 signaling has been implicated in inflammation-induced immunopathogenesis during viral infection (Kayesh et al., 2021), and its suppression by PYC suggests a potential anti-inflammatory mechanism (Verlaet et al., 2019). Mechanistically, PYC inhibits NF-κB activation, leading to decreased transcription of pro-inflammatory mediators such as TNF-α, ICAM-1, and vascular cell adhesion molecule-1, thereby modulating cytokine-driven inflammatory responses and endothelial activation (Matsumori et al., 2007). These findings suggest that PYC may exert its immunomodulatory effects through coordinated suppression of TLR4-mediated NF-κB signaling, although further studies are needed to clarify the underlying molecular mechanisms.

Network pharmacology and protein-protein interaction analyses have identified IL-6, TNF, IL-1B, prostaglandin endoperoxide synthase 2, and NF-κB1 as core molecular targets of PYC, with Kyoto Encyclopedia of Genes and Genomes (KEGG) enrichment highlighting the IL-17 and TNF signaling pathways as key anti-inflammatory mechanisms. These pathway-level predictions were experimentally validated, as PYC significantly suppressed IL-6 and IL-1β expression in lipopolysaccharide-induced BV2 microglial cells, supporting its multi-component, multi-target mode of action (Liu et al., 2024).

Although *in vitro* studies have provided substantial evidence of anti-inflammatory activity, corresponding evaluations using *in vivo* models remain limited. Likewise, clinical trials evaluating antiviral or anti-inflammatory endpoints, such as viral load, disease outcomes, and cytokine biomarkers, are either preliminary or lacking and warrant further investigation. Although PYC has been shown to reduce CRP levels, existing trials are limited by small sample sizes (e.g., five studies totaling approximately 324 participants in a CRP meta-analysis), high heterogeneity (I^2^ ≈ 99%), and variability in dosing regimens, treatment duration, and study populations (Nikpayam et al., 2018).

Similarly, although PYC’s anticancer effects have been demonstrated in multiple cell lines and preclinical models, translational data remain limited. Few clinical studies have evaluated its anticancer efficacy in humans, and mechanistic insights are largely confined to *in vitro* studies. Moreover, standardized dosing, pharmacokinetics, and long-term safety profiles in cancer populations have yet to be comprehensively defined. Addressing these gaps is essential to clarify PYC’s therapeutic relevance and support its integration into evidence-based cancer care.

PYC is generally well-tolerated, with a low incidence of adverse effects (∼1.8%) reported across 70 clinical studies. Most effects are mild and primarily involve transient gastrointestinal discomfort. No serious adverse events, clinically significant cardiovascular effects, or mutagenic, teratogenic, or fertility-related risks have been reported, supporting its favorable safety profile, including in pediatric populations (Verlaet et al., 2017). In a pilot study involving oncology patients receiving chemotherapy or radiotherapy, PYC supplementation (150 mg/day) was associated with a reduced frequency and severity of treatment-related side effects and decreased need for supportive medications. However, potential interactions with the antineoplastic efficacy of chemo- or radiotherapy were not assessed (Belcaro et al., 2008). Therefore, possible drug–nutrient interactions, particularly in cancer and immunocompromised populations, remain insufficiently characterized and warrant careful evaluation in future controlled studies.

Despite its promising antioxidant and immunomodulatory properties, several factors limit the rigorous evaluation of PYC’s therapeutic potential. These include variability in dosing, reported adult dosages range from 30 to 300 mg/day, depending on the clinical indication, formulation, and treatment duration, and the lack of standardized dosing regimens, which complicates cross-study comparisons (Rohdewald, 2002; Belcaro et al., 2013). Low oral bioavailability and interindividual differences in absorption and metabolism of polyphenolic constituents may further influence therapeutic outcomes. Additional challenges include heterogeneity in study design, populations, and outcome measures. Although preclinical and small-scale clinical studies suggest potential benefits, large, well-designed randomized trials are needed to establish safety, optimal dosing, and efficacy, particularly in viral infections and other disease contexts (Bayer and Högger, 2024).

**FIGURE 2:**
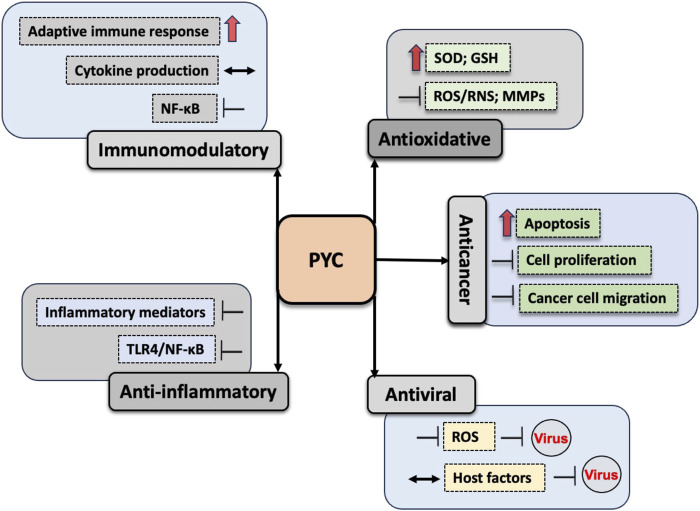
Mechanistic overview of the pleiotropic therapeutic actions of pycnogenol (PYC). This schematic summarizes the principal molecular pathways underlying the antioxidant, anti-inflammatory, immunomodulatory, antiviral, and anticancer effects of PYC. PYC enhances cellular antioxidant defenses by upregulating endogenous antioxidants, including superoxide dismutase (SOD) and glutathione (GSH), while suppressing oxidative stress–associated mediators such as matrix metalloproteinases (MMPs). Through redox regulation, PYC inhibits activation of key inflammatory signaling pathways, notably toll-like receptor 4 (TLR4)– and nuclear factor κ-light-chain-enhancer of activated B cells (NF-κB)–dependent cascades, resulting in reduced production of pro-inflammatory cytokines and mediators. Immunomodulatory effects include attenuation of NF-κB signaling, balanced modulation of cytokine profiles, and enhancement of adaptive immune responses. In cancer-related pathways, PYC promotes apoptosis and suppresses tumor cell proliferation and migration through modulation of oxidative stress and inflammatory signaling. Additionally, antiviral activity is mediated by suppression of excessive reactive oxygen species (ROS), which are required for efficient viral replication, along with modulation of host cellular factors involved in viral life cycles. Red arrows indicate stimulatory effects, black blunt-ended arrows indicate inhibitory effects, and double-headed arrows denote regulatory modulation (either upregulation or suppression), depending on the biological context.

In the near-term, PYC is most likely to serve as an adjunctive or nutraceutical support rather than a standalone therapeutic agent. Evidence indicates it may reduce treatment-related adverse effects and improve quality of life when used alongside conventional therapies, including in chronic inflammatory conditions and oncology settings, provided careful monitoring for drug–nutrient interactions (Belcaro et al., 2013; Rohdewald, 2002). Preventive supplementation could also be considered in populations exposed to oxidative or inflammatory stress, although standardized dosing, long-term safety in vulnerable populations (e.g., cancer or immunocompromised patients), and confirmation of non-interference with primary therapies remain essential. Advancing clinical translation will require well-powered, indication-specific randomized trials, systematic evaluation of pharmacodynamic interactions, and establishment of evidence-based dosing guidelines. These studies are crucial to validate PYC’s *in vivo* antioxidant, anti-inflammatory, immunomodulatory, antiviral, and anticancer effects. The interconnected molecular mechanisms underlying these pleiotropic actions are summarized schematically in Figure 2.

## 7 Conclusion

PYC, a standardized extract of French maritime pine bark, has gained recognition for its broad therapeutic potential. Beyond its well-documented antioxidant effects, PYC exhibits anti-inflammatory, immunomodulatory, antiviral, and anticancer properties, making it a promising candidate for adjunctive management of viral infections characterized by ROS generation, inflammation, and immune dysregulation. The demonstrated activity of PYC against viruses, such as HCV, DENV, and SARS-CoV-2, highlights its broad-spectrum therapeutic potential. The antioxidant properties of PYC are critical for inhibiting ROS production, thereby reducing oxidative stress, a key contributor to viral pathogenesis and chronic inflammation.

Moreover, modulation of key inflammatory pathways and cell signaling cascades suggests that PYC may help mitigate virus-induced immunopathology, suppress tumor cell proliferation, induce apoptosis, and inhibit angiogenesis. Despite encouraging preclinical and limited clinical findings, substantial gaps remain regarding its bioavailability, pharmacodynamics, and efficacy in well-controlled human trials. Therefore, further rigorous *in vivo* studies and high-quality clinical trials are essential to validate its therapeutic potential and clarify its mechanism of action. Continued research on PYC may support the development of novel plant-based antiviral strategies with antioxidant and immunomodulatory benefits.
